# “PrEP’s just to secure you like insurance”: a qualitative study on HIV pre-exposure prophylaxis (PrEP) adherence and retention among black cisgender women in Mississippi

**DOI:** 10.1186/s12879-021-06786-1

**Published:** 2021-10-26

**Authors:** Tiara C. Willie, Mauda Monger, Amy Nunn, Trace Kershaw, Jamila K. Stockman, Kenneth H. Mayer, Philip A. Chan, Adaora A. Adimora, Leandro A. Mena, Deja Knight, Karlye A. Philllips, Stefan D. Baral

**Affiliations:** 1 Department of Mental Health, Johns Hopkins Bloomberg School of Public Health, Baltimore, MD USA; 2 MLM Center for Health Education and Equity Consulting Services, Jackson, MS USA; 3 Department of Psychiatry, Warren Alpert Medical School of Brown University, Providence, RI USA; 4 Department of Medicine, The Miriam Hospital, Providence, RI USA; 5 School of Public Health, Brown University, Providence, RI USA; 6 Department of Social and Behavioral Sciences, Yale School of Public Health, 60 College Street, New Haven, CT USA; 7 Division of Infectious Diseases and Global Public Health, Department of Medicine, University of California, San Diego, La Jolla, CA USA; 8 Harvard Medical School and Harvard T H Chan School of Public Health, Harvard University, Boston, MA USA; 9 Department of Medicine, Gillings School of Global Public Health, University of North Carolina, Chapel Hill, NC USA; 10 School of Medicine, and Department of Epidemiology, Gillings School of Global Public Health, University of North Carolina, Chapel Hill, NC USA; 11 Department of Medicine, University of Mississippi Medical Center, Jackson, USA; 12 Department of International Health, Johns Hopkins Bloomberg School of Public Health, Baltimore, MD USA; 13 Department of Epidemiology, Johns Hopkins School of Public Health, Baltimore, MD USA

**Keywords:** Black women, Cisgender, Pre-exposure prophylaxis, HIV

## Abstract

**Background:**

Pre-exposure prophylaxis (PrEP) has the potential to reduce transmission of HIV among Black cisgender women in the Southern United States (U.S.); however, national data suggests that PrEP initiation is lowest in the South and among Black women compared to other U.S. regions and white women. This study applied intersectionality and PrEP multilevel resilience frameworks to assess how socio-structural and clinical contexts shaped PrEP persistence among Black cisgender women in Mississippi.

**Methods:**

Semi-structured interviews were conducted with eight Black cisgender women in Jackson, Mississippi. This sample was purposively recruited to include PrEP-initiated Black cisgender women.

**Results:**

Six themes identified that shaped PrEP care among Black cisgender women: (1) internal assets, (2) sole responsibility to HIV prevention, (3) added protection in HIV serodifferent relationships, (4) financial issues, (5) trust and distrust in the medical system, and (6) side effects. Black cisgender women reported that PrEP persistence increased control over their sexual health, reduced anxiety about HIV, and promoted self-care. Black cisgender women also indicated that medication assistance programs increased PrEP affordability resulting in continued persistence.

**Conclusions:**

In addition to preventing HIV, PrEP may yield secondary positive impacts on the health and relationships of Black cisgender women. However, very few Black cisgender women in the South are using PrEP given intersectional barriers and thus necessitates adaptive strategies to support PrEP initiation and persistence. Efforts aimed at increasing the coverage of PrEP among Black cisgender women should consider implementation strategies responsive to lived realities of Black women.

## Background

Preventing heterosexual acquisition of HIV among Black cisgender women in the South is a public health priority in the United States (U.S.). Black women are disproportionately affected by HIV compared with women of other racial and ethnic groups [1]. Black women represent 13% of the U.S. women population [2], but account for 57% of all new HIV diagnoses among women [1]. Across the U.S., the South has the highest rates of HIV diagnoses, and Black women account for more than two-thirds of HIV diagnoses among women [3]. These geographic and racial disparities in HIV infection helped inform the U.S. Plan for Ending the HIV Epidemic (EHE). The EHE is an initiative that targets resources to the 57 jurisdictions (e.g., counties, states, municipalities) with the highest burden of HIV which includes the state of Mississippi [4]. Mississippi ranked 7^th^ in the nation for HIV diagnoses [5]. Among women in Mississippi, Black women represented 86% of all new infections and were nine times more likely to acquire HIV than white women [6]. Heterosexual contact is the primary mode of HIV transmission among women and is estimated to account for 87% of all new infections among women nationally [1] and 96% of new infections among women in Mississippi [7]. Enhancing uptake of evidence-based HIV prevention strategies to reduce heterosexual transmission of HIV among Black women, particularly women in the South, is critical.

### State of pre-exposure prophylaxis (PrEP) among U.S. black cisgender women

Pre-exposure prophylaxis (PrEP) is an effective HIV prevention strategy, but there are consistent implementation gaps in PrEP impact among women in the U.S. PrEP currently comprises the use of an FDA-approved daily medication that reduces HIV acquisition among HIV-negative persons [8]. Recent epidemiological data underscores significant racial, gender and geographic-related disparities in PrEP initiation (i.e., receiving a prescription). Specifically, PrEP initiation is lowest in the South compared to other regions [9]. Overall, PrEP initiation is lower among women than men [10], but among the few women using PrEP, PrEP initiation is significantly lower among Black women than white women [11]. Increasing the number of Black women initiating PrEP is critically important because low PrEP initiation directly contributes to low PrEP effectiveness at the population-level (i.e., PrEP’s ability to do more good than harm for a target population in a real-world setting; [12, 13]).

Several studies have described barriers to PrEP initiation among Black women in the U.S. For example, limited PrEP knowledge and awareness can lead to low PrEP initiation [14, 15], and the limited culturally-tailored PrEP marketing compounds the risk of low PrEP initiation [16, 17]. HIV-related stigma [18] and PrEP stigma [19] can contribute to low PrEP initiation due to the negative stereotypes of HIV and PrEP. Also, there are factors related to the healthcare system (e.g., medical mistrust, provider bias) that may negatively impact PrEP initiation among Black women [20].

While addressing barriers to PrEP initiation is key to optimizing the HIV incidence reduction potential of PrEP, so too is enhancing PrEP implementation for marginalized communities such as Black cisgender women. PrEP implementation refers to “the use of strategies to adopt and integrate evidence-based health interventions (i.e., PrEP) and change practice patterns within specific settings” [13]. Individual-level interventions have been developed to increase PrEP awareness among Black cisgender women [21, 22] but few interventions have been designed to address PrEP implementation in clinical settings with the goals of increasing PrEP initiation among this population. Optimizing PrEP implementation in real-world clinical settings is paramount because healthcare providers are the gatekeepers to PrEP (apart from California where pharmacists are allowed to prescribe PrEP; [23]).

Black cisgender women who have initiated PrEP might provide key insights to inform PrEP implementation. PrEP-initiated Black cisgender women may live in the same communities and have shared social experiences as Black cisgender women at-risk for HIV but who have yet to initiate PrEP. Therefore, PrEP-initiated Black cisgender women have firsthand knowledge of the obstacles to PrEP initiation, potential solutions to address those obstacles, in addition to lived experiences navigating the distal components of the PrEP care continuum such as adherence (i.e., how well someone takes PrEP) and retention (i.e., maintaining HIV prevention care, with and without PrEP use) [24]. While qualitative research methods offer an opportunity to assess social processes shaping PrEP persistence from the personal narratives of PrEP-initiated Black cisgender women, there have been few studies investigating these phenomena. Specifically, only two qualitative studies have assessed factors shaping PrEP persistence among U.S. cisgender women [17, 25]. Hirschhorn, Brown [17] found that side effects and provider trust were important considerations for PrEP adherence. Moreover, Park, Taylor [25] discussed positive interactions with providers, insurance coverage, side effects, and pharmacy impediments as barriers and facilitators of PrEP persistence. These studies offer insights into PrEP implementation for U.S. cisgender women; however, this research can be extended to center the experiences of U.S. Black cisgender women. Research that centers Black women’s experiences entails recruiting and asking women about their PrEP experiences within the context of macro-level factors in order to propose upstream strategies to improve PrEP implementation.

### Two frameworks to situate HIV prevention among U.S. black cisgender women

#### Intersectionality

Individual behavior alone does not account for racial- and gender-related disparities in HIV prevention and treatment outcomes [26–29], thus these phenomena are better understood when situated within the context of socio-structural factors. Socio-structural factors, outside of an individual’s control, may impact vulnerability to HIV infection, including access to PrEP. For example, Black women and their communities have experienced violence, discrimination, stigma, and poverty that have disrupted sexual networks, relationship power dynamics and thus thwarted HIV prevention efforts [26, 27]. Despite recent calls to address socio-structural factors and PrEP care [18, 30], few studies have conceptualized Black women’s experiences related to PrEP within an intersectional framework. Historically rooted in Black feminist theory, intersectionality is a theoretical framework that posits macro-level factors (e.g., racism, sexism, classism) produce multiple interlocking sources of oppression and power, which intersect and impact individuals at the micro-level based on their social identities [31–34]. Intersectionality-informed research tends to address the complexities of health research within the context of power and privilege. Research guided by intersectionality can investigate how structural inequities have shaped the lived experiences of marginalized populations and offer potential strategies to address these structural inequities [35].

The historical context of unethical medical practices (e.g., Tuskegee Syphilis Study, Mississippi Appendectomies) and discriminatory experiences within the healthcare system has significantly contributed to medical mistrust in the Black community [36, 37]. Medical mistrust can shape interest and willingness to use PrEP among women [15, 38], such that Black women with strong concerns regarding the trustworthiness of the PrEP medication and healthcare system may be less likely to discuss or use PrEP [20, 39]. High medical mistrust is also related to low PrEP adherence in other diverse populations [40, 41], but *conceptually*, this relationship may also exist among Black cisgender women.

Gendered racism represents the daily gendered racial microaggressions that Black women face due to the simultaneous experience of racism and sexism [42–45]. Gendered racism has yet to be linked to PrEP initiation and persistence, but prior research found that healthcare providers were less willing to prescribe PrEP to Black patients due to biases and stereotypes [46]. Providers who see, evaluate, and treat Black women based on historically-constructed stereotypes (e.g., Jezebel, Sapphire) may reduce PrEP initiation, adherence, and retention among Black women. Black women may also face additional constraints when accessing and being retained on PrEP if healthcare providers perceive Black women as not needing PrEP due to providers’ knowledge of national HIV epidemiological data (19% of HIV infections among women vs. 81% among men).

Inequities related to PrEP initiation, adherence, and retention among Black women may also be shaped by the interlocking nature of racism, sexism, and classism. Economic costs related to PrEP clinical services is an important consideration for women [15, 16, 18, 38], however, structural forces can produce occupational segregation (i.e., systematic concentration of workers in particular jobs; [47, 48]) and the devaluation of Black women’s work (i.e., racial- and gender-related wage gap), which may prohibit PrEP initiation, adherence, and retention. Discrimination related to racism and sexism constrains Black women to low-wage employment in the service industry (e.g., childcare; [49]) and this has long-term impacts on wealth trajectories [50]. Economic inequalities also persist due to the racial- and gender-related wage gap which demonstrates that, on average, Black women make less than both white men and white women [51, 52].

#### Strengths-based approach

Existing research related to PrEP is saturated with research conducted and interpreted from a deficit-based approach as opposed to a strengths- (or asset-based) approach. Deficit-based approaches can identify important needs, but they can also frame communities and individuals as problematic and overlook the positive attributes and assets of health promotion [53]. A recent review on sexual health interventions for Black women posited that asset-based approaches were needed to identify protective factors that help overcome challenging situations and inform interventions that empower Black women [54]. Furthermore, a recent study developed a multilevel model of resilience among PrEP-initiated Emaswati women in real-world settings [55], which offers a solid foundation for future research to explore resilience in relation to PrEP in other contexts, such as Black cisgender women residing in the Southern U.S. Therefore, PrEP implementation that addresses barriers and also enhances protective factors may enhance PrEP initiation, adherence, and retention among Black cisgender women.

In summary, Black cisgender women in the U.S. South are at highest risk for HIV acquisition yet have low rates of PrEP initiation. To address this implementation gap, this study sought to understand multilevel factors influencing PrEP persistence among Black cisgender women residing in the Southern U.S. within an integrated intersectional [31] and strengths-based framework through resilience [55]. Research that underscores a clear understanding of the barriers and facilitators to PrEP persistence will help inform community-based and clinical PrEP programs and interventions tailored to the experiences of Black cisgender women in the Southern U.S.

## Methods

### Study setting

The study was conducted in Jackson, Mississippi. Jackson, the capital of Mississippi, ranked 4^th^ for HIV diagnoses among women in metropolitan statistical areas and had one of the highest rates for HIV-related deaths among women [5]. Jackson is at the epicenter of the HIV hotspots in Mississippi [56]. Black women represented 75% of HIV diagnoses among women [57] despite comprising only 39% of the female population in Mississippi. In Mississippi, women represented only 10% of PrEP users in 2018 [7]. As of June 2020, there were 8 PrEP providers listed with the Mississippi Department of Public Health that offer PrEP services.

### Sample

We recruited and interviewed eight Black cisgender women who had participated in a PrEP program at a clinic that provided preventive and primary care services to diverse populations. At the time of recruitment, there were 12 cisgender women receiving PrEP services at the clinic, and this study purposively sampled Black cisgender women engaged in PrEP. Four cisgender women were excluded from this study because they did not identify as Black. Given the low prevalence of PrEP initiation among cisgender women after initial recruitment at the clinic ended, we conducted preliminary analysis for saturation and determined saturation was achieved. Semi-structured qualitative interviews were conducted with the participants between June 2019 and June 2020. Women were eligible for the qualitative study if they were aged 18 or older, identified as a Black cisgender woman, spoke English, had a PrEP prescription, and provided informed consent.

### Procedures

Women who were prescribed PrEP were invited to complete a semi-structured, qualitative interview. During the semi-structured interviews, participants were asked questions regarding the social, structural, and health system factors related to their experiences during PrEP initiation, adherence, and retention. Analytical memos were created after each interview. Interviews lasted between 45 and 60 min and were audio recorded. Audio files were transcribed verbatim by a HIPAA-compliant transcription company. Participants were remunerated $40. All interviews were conducted by a Black cisgender woman public health researcher trained in qualitative methods. Semi-structured interviews were completed either over the phone or in-person in private rooms. To protect participants’ identity, the researchers changed the names and assigned pseudonyms. The [Institution masked for peer review] IRB approved all study procedures.

### Data analysis

Using a general inductive approach, the semi-structured interviews were coded and analyzed into themes. Open coding was conducted on two interviews by an interdisciplinary team of four coders (including the qualitative interviewer). During the open and axial coding stages, codes were discussed, aggregated and condensed in an iterative process among the research team in order to develop a codebook. The codebook was modified and finalized based on these discussions. All the transcripts were coded based on the finalized codebook by two coders. During research team meetings, there was discussion regarding inconsistencies in code application and interpretation and confirming and disconfirming cases. Qualitative data was coded using Dedoose 4.5 (Hermosa Beach, CA).

### Researcher reflexivity

The first author is a Black cisgender woman from the U.S. South who conducted the interviews and led the coding team. The second author is a Black cisgender woman from Mississippi who was the point of contact with the recruitment site and also assisted with the development of the interview guide. This information was shared with participants during the recruitment phase. Regular participation in team meetings also provided a platform to engage in reflexive discussion regarding prior assumptions and experiences that shaped our interpretation of the findings.

## Results

Table 1 displays the sample characteristics. The majority of the women identified as heterosexual (6/8), were single (6/8) and were employed (5/8). The average age among the participants was 33 years. In terms of PrEP care and retention, five women were currently using PrEP at the time of the interview and three women had discontinued PrEP. Five women had health insurance, which included private insurance through an employer and Medicaid. The majority had last used PrEP less than a year prior. Most of the participants used a PrEP medication assistance program, which significantly reduced the cost of PrEP.

**Table 1:** Sociodemographic Characteristics of PrEP-Initiated Black Cisgender Women

	Full sample		PrEP previous use^a^		PrEP current use^a^	
	*n*	%	*n*	%	*n*	%
Age						
< 35 years old	4	50.0	1	25.0	3	75.0
> 35 years old	4	50.0	2	50.0	2	50.0
Marital status						
Single	6	75.0	1	16.7	5	83.3
Domestic partnership	1	12.5	1	100.0	0	0.0
Separated or Divorced	1	12.5	1	100.0	0	0.0
Employment status						
Full-time	3	37.5	0	0.0	3	100.0
Part-time	2	25.0	1	50.0	1	50.0
Not in the workforce	1	12.5	1	100.0	0	0.0
Unemployed	2	25.0	1	50.0	1	50.0
Monthly income						
Less than US$999	3	37.5	2	66.7	1	33.3
US$1,000-US$1,999	2	25.0	1	50.0	1	50.0
US$2,000-US$4,999	1	12.5	0	0.0	1	100.0
US$5,000 or up	1	12.5	1	100.0	0	0.0
Sexual orientation						
Heterosexual	6	75.0	1	16.7	5	83.3
Lesbian	1	12.5	1	100.0	0	0.0
Bisexual	1	12.5	1	100.0	0	0.0
Place of residence						
House or Apartment	7	87.5	2	28.6	5	71.4
Living in a car/street	1	12.5	1	100.0	0	0.0
Religious affiliation						
Christian	3	37.5	1	33.3	2	66.7
Baptist	1	12.5	1	100.0	0	0.0
Catholic	1	12.5	0	0.0	1	100.0
Nothing	3	37.5	1	33.3	2	66.7
Time since taken PrEP						
1 month or less	5	62.5	0	0.0	5	100.0
7–12 months ago	1	12.5	1	25.0	0	0.0
12–24 months ago	1	12.5	1	25.0	0	0.0
> 24 months ago	1	12.5	1	25.0	0	0.0

*N* = 8. PrEP use (current/previous) is defined by responses to the question “are you currently taking PrEP” Yes = current No = previous

^a^Row percentages

The sections below describe how social, structural, and health system factors influence women’s PrEP persistence. These findings underscore factors that act as barriers to women’s PrEP persistence, but also acknowledge facilitators of these experiences. Overall, we contextualize these factors from an intersectional perspective in order to acknowledge the complexities of Black cisgender women’s lives navigating PrEP care in the Deep South.

### Resilience

#### Internal assets—choice, control and protection of one’s health

Participants felt that PrEP provided them choice and control to protect their sexual health and the use of PrEP had an empowering impact on women’s self-perceptions. For some participants, they felt that an important benefit of PrEP persistence was the added protection against HIV transmission. Chanel stated:

I think of the benefit that it's supposed to be, you know, protect me from HIV.

Also, Tonya described the protection of PrEP like insurance. In particular, she said:

I feel like, it’s protectin’ me. Just like this, I feel like it’s like car insurance. Like, I got car insurance on my car. So, I take my pills to make sure that I know one thing about it. If something ever happens, I ain’t gotta worry about catchin’ that stuff. It’s just to secure you, like insurance. Yeah, it secures you that’s the key, you know, bein’ safe.

The ability to protect oneself from HIV was particularly impactful for participants who experienced familial losses due to HIV-related complications.

I don’t want anything [HIV], so I’m protected. I just wanna be protected. I don’t want this because my auntie died with it, not knowin’ that she had it. [Annamae]

Some participants emphasized the role of PrEP in health prevention behaviors and even viewed taking PrEP as a form of self-care. Nicole said:

It’s a necessity, you know— takin’ care of yourself. You not gon’ have this body forever, but you gon’ have to take care of yourself. It’s like a super Band-Aid that you gotta take every day.

#### Internal assets–reduces anxiety, and promotes relief and happiness

Participants described positive affectivity as a result of actively engaging in PrEP care. In particular, some participants shared that their anxiety and worries regarding HIV infection significantly decreased because of the added protection that PrEP offered them.

I just feel like it’s benefittin’ me. I feel like I don’t have to worry at night. I feel like a new woman. I know that’s how I feel about PrEP. Baby, I feel like a new woman. I’m saved from the damage it could cause somebody. [Tonya]

Similarly, some participants viewed PrEP persistence and retention as a statement of self-love and an opportunity to avoid premature death. Barbara:

I mean, I love myself. I wanna be here long enough to become a grandmother or see them graduate.

Some participants expressed gratitude and happiness in finding and being connected to PrEP clinical services. Jane said “I’m just glad I found PrEP.”

Further, Chanel expressed strong positive affectivity for PrEP that she, as a result, wanted to find a way to encourage other potential PrEP users. She stated:

I am glad that PrEP is around. And even though not as many people who probably could use it are using it, just the fact that we do have the access that the program. I'm on or in, whichever way, has allowed me to be able to be a part of. So, my thought is that I wish it was a way that I could, like say, Hey, Come on in. Come on, y'all.

### Relationship dynamics

#### Sole responsibility to prevent HIV transmission due to partner’s behaviors

Several participants described concerns regarding their partner’s sexual risk behaviors as important motivating factors for their PrEP persistence. For instance, participants often described HIV prevention as their personal responsibility due to the lack of trust with sexual partners. Rudy said:

Like, I really been thinkin’ on stuff. You really have to hit that, ’cause that’s what people don’t think about, you know. They just think, oh, I’m with him. He shouldn’t be with nobody else, and this and that. People don’t think no more. People just think, oh, I’m with this one person, and that. Like, they don’t think. They just put their trust in a man. The way I’ve been hurt and stuff, I don’t put my trust in no man.

Further, some participants expressed concerns that stemmed the lack of communication regarding their partner’s HIV serostatus. For example, Annamae said that her continued engagement in PrEP care was, in part, motivated by her lack of trust in sexual partners disclosing their HIV serostatus. She described her experience as:

I had to tell him I was through. And all he kept sayin’—“I protected you. I protected you,” but, no. “You didn’t protect me because you didn’t tell me.” The idea that he did not tell me the situation, that’s what influenced it. I don’t trust a man at all. We was together three and a half years. He had plenty opportunities to tell me the situation. But he did not, so ain’t nobody—they don’t got it written across their face. “Hey, I got AIDS. I got this, I got that.” They don’t got it on there.

#### Added protection in serodifferent relationship

Participants in serodifferent relationships tended to express positive attitudes towards PrEP adherence and often demonstrated the positive effect PrEP had on sustaining their romantic relationships. Tiffany stated:

It [PrEP] made me feel better. It [PrEP] made me have more confident, and it made me you know, feel like, “Okay, me and my partner can go a long way without any concern.

In addition to reducing anxiety, PrEP persistence in care strengthened relationship satisfaction and functioning. Jane stated:

Did it impact me? Probably because it made us, like, feel safer, so we were, like more calm about it. ’Cause at first, when we started being intimate, you could tell he was just not there. He was freaking out. And then afterwards, he’d freak out. And—you know? He was just going through it. But it made us stronger. And then, you know, just that bonding like going here and knowing everything’s okay. So it helped a lot. I’m actually really glad that there was that option ’cause otherwise, we probably wouldn’t even be together ’cause we’d be fighting or he’d be worrying.

### Health system

#### Financial issues related to PrEP medication and related costs

Participants often discussed the affordability and costs related to PrEP services as a potential barrier but outlined the importance of medication assistance programs. For example, Nicole said:

Um, you know, it was a little mind bogglin’ because I wasn’t sure if all this was gonna be expensive— because I have no insurance and stuff. But she told me there was a coupon that’ll help me pay for the medicine.

Some participants stated that the medication assistance program was the only way they would have been able to engage in PrEP persistence and retention. In particular, Jane stated:

Oh, it’s covered by some Gilead thing. So I have that little card, and man, that medicine’s expensive too. It’s almost like, what, 2000 Or 1000 dollars or something? Like a month? Just crazy. That’s rent for somebody. I know. Right? I’ve been saying— so I’m very thankful for that because—I wouldn’t have even been able to take it in that case.

Some participants also discussed the positive affectivity as a result of having little to no out-of-pocket costs for PrEP.

So, it has been no cost to me, directly outta my pocket. I know that when I went to the clinic, I turned in my insurance information, … I'm happy 'cause right now, I haven't been given a outta pocket amount where I had to, you know, pull the cash outta my pocket. [Chanel]

Participants not only disclosed positive feelings such as happiness, but for some participants, there was even a sense of pride in the fact that they were able to use PrEP and protect themselves at a small cost. Annamae stated:

Hm-mm. Okay. I ain’t even refilled this yet. But, yeah, it’s free. It don’t cost anything.” [shows the receipt] “See, that’s how much it costs right there. That’s how much the pill costs itself, but, see, now I’m payin’ nothin’. See, look where—see how much I pay?” “See, it’s all the way down the line. Zero dollars, 0 cents, 0 dollars, 0 cents.

#### Trust and distrust in the medical system

The positive experience participants had with clinical staff along the PrEP care continuum from learning about PrEP and continuing their PrEP-related services was one of the most significant facilitators to PrEP persistence. Some participants perceived an overall positive, friendly atmosphere at the clinic and also among the staff. For example, Chanel:

I do. I haven't had anything, um, bad to happen. Or if I felt like the doctor or nurses or if they wasn't being compassionate—I can't say that. They're very nice, you know. The people are very compassionate.

Some participants had such a high level of comfort and support from the staff, they perceived the staff to be familial.

At that clinic, they make you feel like family, so I was like, ‘Come on with it. Like, I need to get on that.’ And it’s too much goin’ on out here. [Rudy]

Black cisgender women also discussed their level of comfort and trust with the clinical staff and provided examples of their experiences when accessing PrEP. In Tiffany’s narrative, the clinic nurse normalized conversations on women’s sexual health in order to enhance the comfort level of discussing a potentially sensitive topic. Tiffany stated:

So once I talked to my doctor and stuff, you know, as the conversation went on, I felt more relieved. I felt better, you know…Yes, my nurse that came in and she was talkin’ to me. She was like, ‘We all are women, and we all, you know somewhat experience stuff like that in our lifetime.’ She was like, ‘Don’t feel embarrassed.’ You know, she was just talkin’ to me like a real person—

Some participants described their multifaceted relationship with their doctor because their doctor not only provided medical advice but personal life advice as well. For example, Pearl said:

Cause we talk about everything. Sometimes she might be a counselor. Psychiatrist. She’ll be like, ‘You need to slow down. Kids gonna do this. Kids gonna do that.’ You just have to have trust in your doctors. That’s somebody you need—it’ll be close—this close to your momma.

Interpersonal experiences of gendered racism and discrimination from PrEP clinical staff were not explicitly reported by participants in this study, however some participants reflected on their earlier perceptions of distrust in the medical system. Jane stated:

Just, you know, since a little kid, you’re always afraid of the doctor. Or they’re gonna give you shots, or they’re gonna hurt you. Or— you know, they’re only there to get money. I mean, that’s really not true. Most people that go into this kind of field do it because they wanna help people.

### Side effects

Participants discussed anticipating side effects when starting a new medication, however for some participants, the side effects were particularly debilitating and prevented PrEP retention in care.

I stopped the medication 'cause it was makin' me sick. I ain't gonna say it wasn't helping, but I was havin' all kinds of odd side effects. And I know I wasn't havin' 'em before I started that. Bad stomach pains. Constipation. Palpitation of the heart. I mean, I just stopped. [Barbara]

Similarly, another participant shared her difficulty with the side effects of PrEP. Rudy stated:

I don’t know what it was about Truvada, but it just had me sick. It was just, like, havin’ a morning sickness or something. Like, you couldn’t really keep nothing down for it. Truvada just, I don’t know, but that was just rough on me.

## Discussion

Optimizing PrEP implementation presents an opportunity to leverage biomedical advances to curb HIV incidence. However, there has been very limited implementation study among U.S. Black cisgender women. Our study adds to a growing body of evidence on PrEP persistence in real-world settings, specifically among U.S. Black cisgender women. Extant qualitative research among U.S. cisgender women have described several factors shaping PrEP persistence such as provider trust, side effects, and costs [17, 25]. Factors related to the health system are important to address, and our study extends current research by describing how the socio-structural context further complicates experiences of PrEP persistence among Black cisgender women. For example, our findings from women’s narratives indicate that resilience (i.e., having personal control over sexual health, reduced anxiety), relationship dynamics (i.e., sole responsibility for HIV prevention, added protection in serodifferent relationships), and health system factors (i.e., financial issues, medical mistrust, and side effects) may either promote or impede adherence to PrEP and staying in PrEP care. Using an intersectional lens, there are interlocking systems of power and oppression (e.g., racism, sexism, classism) that can constrain Black women’s engagement in PrEP care, but some women demonstrated resilience and have identified positive impacts of PrEP that help them remain engaged in care.

Consistent with previous research with PrEP-initiated women in Eswatini [55], Black cisgender women in this study discussed internal assets and resources that aided in the promotion of resilience while engaging in PrEP clinical services. Black cisgender women’s ability to endure socio-structural challenges and access PrEP in order to reduce HIV acquisition is a potential manifestation of resilience. After accessing PrEP clinical services, Black cisgender women in this study emphasized the importance of having control over their sexual health in addition to positive affect such as happiness, reduced anxiety, and gratitude for being in PrEP care. Some Black cisgender women also perceived PrEP care to be synonymous with self-love and self-care, which is contrary to racialized gender depictions of Black womanhood such as the Silencing Self [58] and the Strong Black Woman Schema [59]. The Silencing Self suggests that women inhibit self-expression in order to maintain relationships and avoid retaliation, loss, or conflict [58]. The Strong Black Woman Schema is a positive cultural ideal that depicts Black women as resilient, independent, and strong while maintaining multiple social roles (e.g., mother, provider) [60, 61]. The Silencing Self is an important component of the Strong Black Woman Schema [62], as Black cisgender women minimize their personal needs in order to care for others and as a result, self-care practices are often underutilized. Despite being developed within the context of oppression and unequal power dynamics [60, 61], some of these depictions are positive (e.g., Strong Black Woman Schema) but may still lead to undesired health outcomes [62–64]. Our findings indicate that Black cisgender women using PrEP may find a way to balance these competing demands and socially constructed manifestations of their identities to prioritize their “self-care” behaviors. It is also possible that striking this balance may be difficult to achieve and have program implications (e.g., low PrEP initiation among Black cisgender women). Altogether, a culturally-responsive empowerment-based intervention that centers women’s personal strengths within the broader socio-structural context may create a supportive environment that facilitates PrEP initiation and persistence.

The role of trust and individual vs. collective responsibility for HIV prevention in Black cisgender women’s sexual relationships is an important facilitator of PrEP persistence. Consistent with previous research among PrEP-initiated Emaswati women in real-world clinics [55], Black cisgender women in serodifferent relationships described how being engaged in PrEP improved their communication and relationship with their partner. Women in serodifferent relationships in long-term partnerships reported greater trust and reduced anxiety regarding HIV acquisition; and a greater sense of collective responsibility with their partner in terms of preventing HIV. In contrast, Black cisgender women in dating relationships reported distrust of male sexual partners and felt that it was their individual, possibly sole, responsibility to prevent HIV acquisition. Distrust of male sexual partners often stemmed from concerns around infidelity in the relationship and partners’ non-disclosure of HIV seropositive status. While women’s awareness of these potential risk factors can encourage their PrEP adherence, it can also be psychologically burdensome for Black cisgender women to assume sole responsibility for preventing HIV in their relationships [65] in addition to managing sociocultural expectations of racialized gender roles such as being caretakers of the Black community [64, 66]. One way to potentially alleviate the societal pressures and sole responsibility of HIV prevention is to reframe public health messages towards collective action [65]. For example, health communication messages could display open communication about PrEP with your partner or other community members. Public health efforts aimed at promoting PrEP among Black cisgender women could consider community mobilization strategies that reach Black women and their sexual networks and focus on a collective responsibility to fight HIV infection by improving PrEP initiation and adherence.

Women described barriers and facilitators they experienced in clinical encounters as Black cisgender women, which included the affordability of PrEP and related costs; having trust and rapport with clinical staff; and side effects of the medication. Side effects is an important contributor to delays in PrEP initiation[17, 18] and discontinuation [67]. Starting conversations early with women could assuage concerns regarding side effects [68]. Women’s perceptions of their clinical experience with PrEP should be also viewed within the context of living in Mississippi, and the interlocking systems of power (e.g., racism, sexism, classism) that they have to navigate. Socio-structural factors can constrain Black women’s economic self-sufficiency [69], which could inadvertently curb women’s ability to afford PrEP and its related costs, and/or navigate the health care system. In particular, in Mississippi, there is no equal pay law [70] and Black women make 55 cents to every 1 dollar made by a white man [71]. These socio-structural factors contribute to the increased likelihood of poverty among Black women in Mississippi [52, 72]. As such, participants consistently shared the importance of being a part of the patient medication assistance program, which significantly reduced the costs related to PrEP care. Similarly, limited financial reserves for PrEP was noted as a common reason for discontinuing PrEP care among Black women. When paired with an opt-out process, patient medication assistance programs have the potential to combat structural interlocking systems of power (e.g., racism, sexism, classism). Specifically, an opt-out process in all PrEP-prescribing settings (e.g., primary care, infectious disease) would allow all Black women to be assessed for eligibility of these medication assistance programs and increase affordability of PrEP. There is also an opportunity to increase community knowledge of these medication assistance programs, and possibly leverage patient advocacy to help with facilitation. However, patient assistance programs may be unable to sustain PrEP requests from individuals without insurance, as a result additional advocacy for Medicaid expansion is important in states like Mississippi in order to increase access to PrEP [73].

An important facilitator of PrEP persistence in care was having a positive relationship with the clinical staff. Women often discussed the positive experiences they had during the PrEP clinical encounter. For example, Black cisgender women discussed having PrEP counselors and clinicians normalize positive conversations on sexual health, and also thoroughly explain the process of accessing PrEP. More importantly, some Black cisgender women described their relationship with the clinical staff as familial, which coincides with the Afrocentric concept of communalism [74–76]. Creating fictive kinships with clinical staff may help Black cisgender women develop a sense of belonging, community, and stronger bonds with trusted community members such as healthcare providers.

This finding is interesting in light of the compelling research linking the context of structural racism and perceptions of medical mistrust [37] as well as the link of medical mistrust among Black cisgender women and PrEP discussions with providers [20]. The narratives from Black cisgender women in the current study discussed positive trusting relationships with clinical staff, which may have buffered the potential negative effects of medical mistrust on PrEP persistence. Our findings regarding trust with providers are consistent with other studies on PrEP retention among cisgender women in Chicago [17] and New York [25]. Together, our findings coupled with existing research with PrEP-initiated women, support the importance of increasing and maintaining trusting relationships between clinicians and Black cisgender women in order to enhance experiences in PrEP persistence.

Some study limitations merit consideration. Central to the public health challenges of this research is that there are not many Black cisgender women engaged in PrEP in the South. Thus, while this study interviewed the majority of Black cisgender women engaged in PrEP care services at the recruitment site the sample remains small. The few published studies that examined PrEP persistence and retention in real-world settings among U.S. cisgender women recruited similar numbers of Black cisgender women [17, 25]. Despite the small sample, we achieved saturation on a diverse array of themes central to the PrEP implementation strategies for Black women in the South. To determine the transferability of our findings to other similar contexts [77], thick descriptions regarding the context of the original research were provided to help other researchers make the final judgement on how transferrable these findings will be to their unique context. In this study, PrEP-initiated Black cisgender women were purposively recruited and sampled. Purposeful sampling strategies is very common in qualitative research and allows for the “identification and selection of information-rich cases.” (78) Therefore, the goal of the current research was to provide an expressive and reflective account of the phenomenon of interest (i.e., factors influencing PrEP persistent among PrEP-initiated Black cisgender women in the U.S. South) and not to ensure generalizability. Moreover, there were synergies in the themes explored here with studies of Black women from other parts of the US.

## Conclusions

Black women across the South continue to be disproportionately affected by HIV highlighting unmet needs. Addressing these needs requires more research and programmatic initiatives that address PrEP initiation and persistence among Black cisgender women but situated within realistic socio-structural context. As national and state HIV prevention efforts continue to enact the Ending the HIV Epidemic Plan, it will be important to acknowledge, understand, and be responsive to the needs and lived experiences of Black cisgender women. National and state efforts to enhance PrEP initiation, adherence, and retention should leverage PrEP facilitators such as empowering Black women and training clinical staff to offer culturally-responsive care. In addition to boosting facilitators, public health efforts should consider addressing barriers to PrEP persistence, such as assessing eligibility for a medication assistance program among all Black women, and potentially collaborating with other community-based organizations to reframe HIV prevention from a collective action perspective. Due to structural racism, medical and provider mistrust, PrEP initiation and persistence might be difficult to achieve among Black cisgender women. Thus, it is critically important to promote research on PrEP initiation and persistence in trusted, non-clinical settings that Black cisgender women may frequent. Trusted, non-clinical settings might offer an additional avenue for Black cisgender women to circumvent specific PrEP barriers, while also providing some alternative safe spaces to engage in women-controlled HIV prevention care.

## Data Availability

The data that support the findings of this study are available to the investigative team but are not publicly available.

## Abbreviations

PrEP: Pre-exposure prophylaxis
HIV: Human Immuno-Deficiency Virus
U.S.: United States
IRB: Institutional Review Board
HIPAA: Health Insurance Portability and Accountability Act
